# Somebody to Lean On: Community Ties, Social Exchange, and Practical Help during the COVID-19 Pandemic

**DOI:** 10.1177/15356841231159370

**Published:** 2023-03-22

**Authors:** Martin Horak, Shanaya Vanhooren

**Affiliations:** 1 University of Western Ontario, London, ON, Canada

**Keywords:** community ties, social exchange, practical help

## Abstract

During a community-wide crisis, practical help from others in the community can allow individuals to manage a variety of extraordinary household needs. In this article, we synthesize insights from research on disaster resilience, social support, social networks, and social exchange into a theoretical model of factors that shape individual access to help beyond the family. We suggest that community ties—local neighborhood, associational, and friend relationships—are significant avenues for accessing help and that helping behaviors in the community are structured by social exchange. We test this model in the early months of the COVID-19 pandemic, drawing on a survey of 4,234 Canadians and Americans. We find that all three kinds of community ties significantly increase the likelihood of receiving and giving help; that there is a strong, positive two-way correlation between giving help and receiving help; that relationships between community ties and helping behaviors are mediated by social exchange; and that individuals in extraordinary need tend to both receive and give more help than others. Our findings provide broad-based evidence for the importance of local social ties and social exchange processes in structuring access to practical help in times of extraordinary need.

## Introduction

When a community experiences a major external shock such as a natural disaster or a pandemic, individuals and households often turn to others in their community for help and support (LaLone 2012; Norris et al. 2008). In the face of widespread economic, social, and (in some cases) infrastructural disruption, the resources of governments and other formal organizations are strained (Aldrich and Meyer 2015; Carlsen, Toubøl, and Brincker 2021; Kyne and Aldrich 2020), heightening the reliance of individuals and households on others in the community to meet pressing and unexpected needs.

In this article, we draw together insights from the literatures on disaster resilience, social support, community ties and social exchange to propose a model of factors that shape individual access to practical help in the community in times of crisis. We test this model in the context of the early months of the COVID-19 pandemic, drawing on data from a survey of 4,234 Canadians and Americans fielded between August 25 and August 31, 2020.^1^ “Practical help,” as we conceive of it, includes a variety of forms of instrumental support that range from cooking meals and running errands to lending tools and supplies. Research on disaster recovery and resilience has shown that such instrumental support is vitally important to well-being in crisis conditions (Aldrich and Meyer 2015; Tobin-Gurley, Peek, and Loomis 2010), but there is much that we do not know about the factors that shape individual and household access to practical help.

While family are a primary source of support in crisis (Cross et al. 2018; Hurlbert, Haines, and Beggs 2000), social relationships beyond the family are also significant. Many people do not live near extended family; even when they do, family members may not have the necessary resources or may not be willing to offer needed support (Klinenberg 2003). As a result, for many individuals and households, broader social connections are critical for coping and recovery (Aldrich and Meyer 2015; LaLone 2012; Norris et al. 2008). Summarizing findings from the literature, Gauthier et al. (2021) noted that “being socially connected is one of the best predictors of resilience during natural disasters” (p.e89). Our own survey data showed that 33 percent of respondents received practical help from family only during the COVID-19 pandemic, 25 percent received help from non-family only, and 42 percent received help from both.^2^ Social ties outside the family are thus an important part of the “ensemble” of connections that offers “stable and adaptive support” to individuals and households in crisis (Wellman and Wortley 1990:558).

Our model of factors that shape access to practical help beyond the family focuses on three main elements: community ties, social exchange, and need. Disaster resilience research has found that interpersonal help in times of crisis rarely comes from strangers; rather, it flows through existing social relationships in the local community (Murphy 2007; Perry, Hawkins, and Neal 1983), which we call “community ties” (Wellman and Wortley 1990). Building on existing research across several fields, we theorize that certain kinds of community ties may be particularly significant sources of practical help: relationships with neighbors, relationships with individuals in community associations, and relationships with close friends. Following Wellman and Wortley (1990), we suggest that each kind of relationship provides access to distinct kinds of help, so they are all independently valuable as potential sources of help in crisis.

How—that is, through what social mechanisms—do community ties provide access to help? While literature on interpersonal help in crisis times tends to analyze giving and receiving separately, there is empirical evidence that they are strongly correlated (Kaniasty and Norris 1995b). Drawing on insights from social support, disaster resilience, and social exchange research, we theorize that helping behaviors beyond the family are structured by processes of social exchange and that giving and receiving tend to be two facets of the social exchange of help. Community ties, therefore, provide access to help in a crisis because they are venues for the ongoing social exchange of help.

This exchange-based perspective on helping behaviors has implications for the relationship between need and the receipt of practical help. Individuals who are experiencing extraordinary need are more likely to receive help than those who have less urgent needs, either because they ask for help (Hurlbert et al. 2000), or because others see their need and offer help (Blokland 2017). However, insofar as helping behaviors are structured by social exchange, receiving help may depend on having helped others in the past, and/or it may motivate those who have received help to help others in turn. Access to help in times of need may thus be connected to one’s own helpfulness to others in the community. While our theoretical model is grounded in existing findings in a variety of literatures, there is no work that we are aware of that draws on a large and geographically widespread survey sample to empirically investigate community ties and social exchange as correlates of helping in a time of crisis.

The COVID-19 setting can tell us much about the factors that shape access to practical help in a community-wide crisis. Most of the existing research on community-based action in crisis has focused on natural disasters. Disasters damage or destroy physical infrastructure, so analysis has tended to focus on community capacity for collective action, rather than on individual-level help (Aldrich 2012; Aldrich and Meyer 2015; Norris et al. 2008; Pfefferbaum et al. 2005; Sherrieb, Norris, and Galea 2010). The COVID-19 pandemic, by contrast, did not damage physical infrastructure. Nonetheless, the combination of the virus itself and the public health response produced a cascade of widespread disruptions that amounted to a community-wide crisis. Many households lost their income, daily schedules were upended with school and workplace closures, caregiving arrangements for children and the elderly were disrupted, and securing even basic amenities became difficult for some vulnerable populations.

Such disruptions were particularly acute during the early months of the COVID-19 pandemic, when lockdowns and closures were widespread, vaccines had not yet been developed, and individuals had not had time to develop coping strategies. It was a time during which practical help in the community took on a heightened significance for many, and anecdotal reports of people helping each other in many ways emerged. Such reports provided the initial impetus for the development of the present research. Descriptive results from our own survey (Figure 2) support the idea that helping behaviors outside the family were widespread in the early months of the pandemic, with over half of respondents reporting they had given practical help to someone outside their family, and nearly half saying that they had received such help.

Compared with most natural disasters, the social and economic disruptions that accompanied the pandemic were very geographically widespread. Quite suddenly, millions of individuals and households across North America (and the world) had to cope with multiple disruptions to their lives. As a result, we can investigate the factors that shape access to practical help in a crisis on a very broad scale, drawing on nationally representative (United States and Canada) survey data that allows us to control for a variety of demographic and contextual variables. In this article, we test a series of hypotheses that address the following key questions flowing from our theoretical model: (1) How do community ties with neighbors, with friends, and through associations affect the likelihood of receiving and giving practical help?; (2) To what extent are giving and receiving help structured by social exchange?; and (3) Are people who face extraordinary need at a time of community-wide crisis more likely to receive help than others?

Using survey-weighted generalized linear modeling, we find that neighborhood, associational, and friendship ties are all positively associated with both receiving and giving help. We find that these associations hold for each kind of community ties when controlling for the other kinds, suggesting that they are complementary facilitators of access to help. We also find a strong direct association between giving and receiving help when controlling for a wide range of other factors. Probing further through mediation analysis, we find that relationships between all three forms of community ties and helping behaviors are significantly mediated by social exchange. Finally, we find that those in extraordinary need receive more help than others, but that in certain cases they also *give* more help than others. Taken together, our results provide robust empirical support for the importance of community ties and social exchange processes in shaping access to practical help outside the family in the context of a community-wide crisis.

## Community Ties and Help: What Kinds of Relationships Matter?

Community ties are locally based social relationships beyond the household and family. Everyone has a unique network of such relationships. An individual’s access to social support of many kinds is influenced by the structure of their local social relationships (House, Umberson, and Landis 1988; Wills and Ainette 2012). While the ubiquity of online communication means that many individuals’ social networks now reach far beyond the local scale (Quan-Haase, Mo, and Wellman 2017), most practical helping behaviors depend on physical proximity, and therefore on distinctly local ties (Haines, Hurlbert, and Beggs 1996). Research on disaster resilience suggests that an individual’s local network is an important source of practical help in the wake of a natural disaster (Aldrich 2012; Aldrich and Meyer 2015; Hurlbert et al. 2000; Murphy 2007; Norris et al. 2008; Perry, Hawkins, and Neal 1983; Pfefferbaum et al. 2005). According to Aldrich and Meyer (2015), “networks provide access to various resources in disaster situations, including information, aid, financial resources, and childcare along with emotional and psychological support” (p. 256). Individuals who are socially isolated, or who have been displaced from their local network, are much more likely to struggle (Klinenberg 2003; Tobin-Gurley et al. 2010). At the same time, network embeddedness—and therefore access to support in disaster situations—is uneven, both across communities (Elliott, Haney, and Sams-Abiodun 2010) and within them (Aldrich 2011).

While there is a consensus among researchers that local social ties are important for access to help in crisis situations, we have a limited understanding of which kinds of ties within an individual’s network matter, and how. Much of the research on disaster situations conceptualizes local social networks broadly as a component of “social capital”—a multifaceted concept that also includes norms (Sherrieb et al. 2010:234) and can be collective or individual (Wu 2020). A few researchers have examined practical help in disasters through the theoretical lens of social support. Their work tends to focus on the significance of “strong ties” (relationships with family and friends, characterized by strong affective attachment) versus “weak ties” (more affectively distant relationships with neighbors, acquaintances, work colleagues and others). The research is limited, and the findings are mixed. For instance, a recent study found that strong ties were more important to meeting individual support needs than weak ties during the COVID-19 pandemic (Carlsen et al. 2021), but an earlier study of practical help in a disaster found that weak ties were more frequently used for help than strong ones (Hurlbert et al. 2000).

To understand better which kinds of community relationships provide access to help and how, we make two conceptual and theoretical moves. First, we move beyond the strong/weak ties dichotomy that is common in disaster resilience work; and second, we build on work in community sociology to emphasize the importance of variety in an individual’s local social network. The concept of “weak ties” includes many kinds of relationships—with neighbors, work colleagues, association-based acquaintances, and others—that might provide access to different kinds of help. Meanwhile, strong ties include both family and friends, so the concept cuts across the family/non-family distinction. As we will discuss further below, there is evidence from prior research that helping dynamics are different among family than among friends, even though both are considered “strong” ties. Given these analytical limitations of the strong/weak ties dichotomy, we focus instead on more concrete categories of local social ties, highlighting three that previous research has identified as conduits for practical help in crisis: connections with neighbors, connections through local associations, and relationships with close friends.

We focus on neighbors for two main reasons. First, the neighborhood has long been recognized as a significant site of social support (Henning and Lieberg 1996; Wellman and Wortley 1990; Wethington and Kavey 2000), and neighbors have been shown to be common sources of practical help in disasters (Kyne and Aldrich 2020; Murphy 2007). Second, many forms of practical help (such as lending supplies or running errands) benefit from close physical proximity, which may make neighbors a particularly valuable source of help.

Local associations—ranging from religious organizations and recreational organizations to service clubs and ethnic associations—are seen as important in the disaster recovery and resilience literature mainly because they facilitate bottom-up, community-based collective action, which can provide basic services in the wake of widespread infrastructure damage (Aldrich 2012; Aldrich and Meyer 2015). However, formal organizations are also venues in which individuals develop interpersonal relationships that they can call on for support (Small 2009). Most local associations operate at a broader spatial scale than the neighborhood; as Kyne and Aldrich (2020) argued, they may thus facilitate “bridging” social ties, which extend the reach of an individual’s network, broadening their access to resources (p. 63).

Relationships with neighbors and people in local associations tend to be weak ties. By contrast, close friendships are, by definition, strong ties, characterized by significant affective attachment. In times of crisis, close friends may be willing to provide more resource-intensive or time-intensive forms of help than an individual could get from their weak-tie relationships (Hurlbert et al. 2000; Wellman and Wortley 1990). Any one individual typically has a small number of close friends. Indeed, as our descriptive survey data confirm (see online Appendix B, available at https://doi.org/10.5683/SP3/UB9P9P), a significant minority have no close friends at all in the local community. However, the strength of affective bonds may make even one or two close friends a valuable source of practical support in crisis.

Because they are distinct in terms of their geographical reach, membership, and the strength of affective bonds, each of these three kinds of community ties can provide access to different sets of resources, and different kinds of help. As Wellman and Wortley (1990) noted, having a varied network of community ties that includes different kinds of relationships with different people gives individuals access to “stable and adaptive” social support (p. 558). Not all individuals have such diverse networks, of course, and the degree to which an individual’s network is characterized by multiplex ties (Kuwabara, Luo, and Sheldon 2010)—that is, by relationships with people who are at once friends, neighbors, and co-members of community groups, for instance—also varies (see also Greenbaum 1982; Guest and Wierzbicki 1999). As we discuss later, our empirical survey data show weak correlations among neighborhood, associational, and friendship ties, suggesting that many individuals in our sample have some kinds of community ties but not others. Nonetheless, since each kind of community tie can play a distinct role in facilitating access to help in crisis, we expect that all three kinds are separately important as sources of access to help in a community-wide crisis.

## Social Exchange, Helping Behaviors, and Need

Most studies of helping behavior in crisis focus either on giving (Haines et al. 1996; Murphy 2007) or on receiving (Carlsen et al. 2021; Hurlbert et al. 2000; Kaniasty, Norris, and Murrel 1990), not on both. However, the two are necessarily related, of course, since any helping act involves both giving and receiving. As we have seen, most helping behavior in crisis situations takes place within existing relationships, rather than among strangers, making it likely that giving and receiving help are connected to each other through relational mechanisms. Understanding the relational mechanisms that underpin giving and receiving help is thus important, as it may have implications for the conditions under which individuals in need can access help from others.

One strand of research on help-giving in disasters emphasizes community solidarity and altruism as drivers of interpersonal helping (Aresi et al. 2022). In the wake of natural disasters and other shocks to a local community, there is often an initial “outpouring of immediate mutual helping in affected areas” (Kaniasty and Norris 1995a: 450). This groundswell in informal help-giving has been documented in many crisis settings, including in the context of COVID-19 (Aresi et al. 2022; Carlsen et al. 2021). Some researchers interpret this as the result of a temporary but powerful community solidarity that emerges in the face of mass adversity, which produces an “altruistic community” marked by extraordinary levels of other-regarding behavior (Giel 1990) and “catastrophe compassion” (Zaki 2020).

There is reason to believe, however, that post-disaster help-giving is not driven only—or even primarily—by altruistic giving born of an extraordinary community solidarity. One of the few large-N studies of helping behaviors after a natural disaster found that those who received help from others were also more likely to give it (Kaniasty and Norris 1995a). The same relationship has also been documented in the broader literature on social support (Bowling, Beehr, and Swader 2005; Jung 1990). This association between the likelihood of giving and receiving in real-world settings suggests that helping behaviors may be structured at least in part by social exchange. Indeed, experimental work has found that while altruistic support is common within family units (Rachlin and Jones 2008), among non-kin social support is significantly exchange-based, even for close friends (Curry, Roberts, and Dunbar 2013).

Social exchange theorists identify four kinds of social exchange (see Molm 2001, and Lawler, Thye, and Yoon 2008, for reviews). Negotiated exchange occurs when two individuals exchange things of value simultaneously through mutually agreed terms. Reciprocal exchange involves a relationship over time, in which individuals trade favors with one another. Generalized exchange

is the practice of giving something of benefit to another person without them giving something back in return (e.g., helping a stranded motorist), because one expects to receive benefits from someone else in the future or because one has received benefits from someone else in the past. (Whitham 2021:503–504)

Finally, productive exchange involves multiple individuals pooling their resources for shared benefit. While the collective action needed to cope with widespread infrastructure damage in natural disasters mobilizes productive exchange relationships, we suggest, following Molm (2010:126), that practical interpersonal help typically mobilizes reciprocal or generalized forms of exchange.

There is extensive research and debate concerning the psychosocial mechanisms that motivate individuals to engage in reciprocal or generalized exchange. Some accounts, anchored in the work of Gouldner (1960) and supported by both experimental and natural-setting research, emphasize the importance of the norm of reciprocity, such that individuals seek to return help received insofar as they believe that it is the “right” thing to do (Uehara 1995; Whitham 2021). Other accounts emphasize the expected utilitarian benefits of returning favors given (Liang, Krause, and Bennett 2001). There is also substantial empirical support for esteem-based explanations of reciprocal exchange, which suggest that “the giving of help by vulnerable people releases them from their position of dependence, increases their self-confidence and reduces social isolation” (Bredewold, Tonkens, and Trappenburg 2016:538). Indeed, studies of help among socially disadvantaged individuals have found that individuals are reluctant to ask for help unless they can return the favor (Bredewold et al. 2016; Nelson 2000). Furthermore Molm, Collett, and Schaefer (2007) have found that the practice of generalized exchange can be self-reinforcing since it leads to an increase in solidarity—that is, individual identification with the group. In this sense, then, exchange-based helping and altruistic helping in community crisis may, in fact, be causally linked.

While investigating the psychosocial mechanisms that motivate reciprocal and generalized exchange is beyond our scope, the important point is that there are numerous plausible reasons to expect that the giving and receiving of interpersonal help are causally connected through social exchange. For this reason, we suggest that it is useful to conceptualize giving and receiving of interpersonal help in the community not as discrete phenomena, but rather as two facets of exchange-based *helping behaviors.* Helping behaviors can be structured through reciprocal (or “direct”) exchange, where two individuals exchange help over time, or through generalized (or “indirect”) exchange, where individuals receive help from some, and “pay it forward” by giving help to others (Molm 2001; Whitham 2021). Indeed, we expect that helping behaviors in the community most likely involve both forms of exchange, such that individuals simultaneously exchange help both directly and indirectly with others in their social network. This combination of direct and indirect exchange may give individuals access to a flexible set of helping resources in times of need.

An exchange-based perspective on helping behaviors has implications for the relationship between giving and receiving help. If receiving help is part of a broader dynamic of reciprocal and generalized helping exchange, there should be an individual-level correlation between the incidence of receiving help and the incidence of giving help. Furthermore, we would expect the correlation to exist in both directions, such that those who give help are more likely to receive it, and those who receive help are more likely to give it. In an exchange-based helping dynamic, in other words, accessing help may be to a significant extent a function of an individual’s past record of giving help to others, and/or of their ability to provide help to others in return.

Insofar as practical help is exchange-based, this should also affect the relationship between individual need and helping behaviors. The literatures on social support and disaster resilience both suggest that individuals and households facing extraordinary need mobilize their social networks by asking for help (Hurlbert et al. 2000; Kaniasty and Norris 1995b; Wellman and Wortley 1990). However, insofar as help is exchange-based, mobilizing support from other people may be contingent on past, present, and/or future ability to provide support to others. Access to help in need, in other words, may come with the social “cost” of being helpful.

## Community Ties, Social Exchange, and Practical Help: Model and Hypotheses

**Figure 1.**
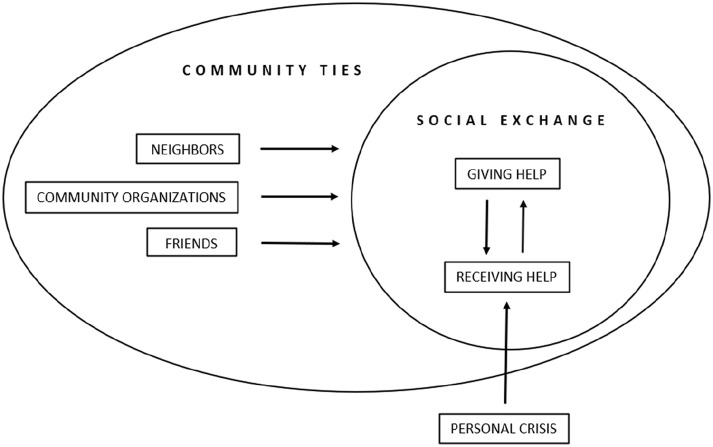
Community ties, social exchange, and practical help.

Figure 1 synthesizes what we have discussed into a theoretical model of factors that shape individual access to help. Access to practical help beyond the family is shaped by an individual’s network of community ties, represented by the large oval. Community ties are composed of many kinds of relationships, which may include relationships with friends, co-members of community groups, neighbors, and/or others. These relationships provide access to help in times of need because they are venues in which individuals practice the ongoing social exchange of help, represented by the smaller oval. Community ties are venues for direct and indirect exchanges of help and support with others, thereby laying the relational groundwork for accessing help in need. Different kinds of relationships provide access to distinct resources, so ties through neighbors, community organizations, and friends are each separately valuable sources of access to help. Those who are experiencing personal crisis can “activate” their social network by asking for help, or they may receive help unasked from others in their network who see their need, but because of the exchange-based character of helping behaviors, accessing help through community ties is contingent on being helpful to others as well.

Based on this model, we propose a series of hypotheses regarding the correlates of practical helping behavior outside the family, which we test on a survey sample of respondents from across North America in the early months of the COVID-19 pandemic. In contrast to studies that use detailed information from a limited number of respondents to directly map individual social networks and patterns of helping (e.g., Hurlbert et al. 2000), our work draws on data from a survey of over 4,000 respondents to identify statistically significant correlates of helping behaviors at the aggregate level across the United States and Canada.

Our first set of hypotheses concerns relationships between different kinds of community ties and access to help. As noted earlier, we theorize that ties with neighbors, ties developed through community associations, and ties with friends all provide significant access to help, so we expect individuals who have any of these kinds of relationships to be more likely to receive help from outside the family than those who do not. Furthermore, since we posit that each kind of community tie facilitates access to different resources and kinds of help, we expect that each kind of tie will be separately significant when accounting for the other kinds. Finally, since receiving help requires someone to give it, insofar as giving occurs within networks of community ties, we also expect that these community ties will also be channels through which help is given to others. Our hypotheses are as follows:

**Hypothesis 1 (H1):** Individuals who know their neighbors are more likely to receive help from people outside their family during COVID-19.
**Hypothesis 2 (H2):** Individuals who know their neighbors are more likely to provide help to people outside their family during COVID-19.
**Hypothesis 3 (H3):** Individuals who are members of community organizations are more likely to receive help from people outside their family during COVID-19.
**Hypothesis 4 (H4):** Individuals who are members of community organizations are more likely to provide help to people outside their family during COVID-19.
**Hypothesis 5 (H5):** Individuals who have a close friend in the community are more likely to receive help from people outside their family during COVID-19.
**Hypothesis 6 (H6):** Individuals who have a close friend in the community are more likely to provide help to people outside their family during COVID-19.

Our second set of hypotheses tests for the social exchange foundations of helping behaviors. If giving and receiving help outside the family are structured by social exchange, then those who give help should be more likely to also receive it, and vice versa. Furthermore, since individual social networks are composed of multiple kinds of ties, this positive relationship between giving and receiving should exist even when controlling for particular kinds of community ties. Our specific hypotheses are:

**Hypothesis 7 (H7):** Individuals who help people outside their family during the COVID-19 pandemic are more likely to receive help from others outside their family.
**Hypothesis 8 (H8):** Individuals who receive help from people outside their family during the COVID-19 pandemic are more likely to help others outside their family.

As discussed, we theorize that ties with neighbors, friends, and people in community associations provide access to help because they are venues in which individuals exchange help on an ongoing basis. The effects of all three kinds of community ties on receiving help should thus be contingent on the giving of help, and vice versa. We thus further hypothesize that:

**Hypothesis 9 (H9):** The association between community ties and receiving help is mediated by social exchange and is thus contingent on an individual also giving help to others.
**Hypothesis 10 (H10):** The association between community ties and giving help is mediated by social exchange and is thus contingent on an individual also receiving help from others.

Our final set of hypotheses concerns the relationship between need and help. As we noted above, the literature on disaster resilience shows that individuals who experience extraordinary need mobilize their social networks to ask for help. Since there are many forms of extraordinary need, we focus on two that were significant and clearly measurable in the COVID-19 context: having a case of COVID-19 in the household and experiencing financial hardship due to pandemic disruptions. We expect that individuals who experienced either of these were more likely to receive help. However, insofar as help is exchange-based, we also expect individuals who got sick or experienced financial hardship to be more likely to help others. Our hypotheses are as follows:

**Hypothesis 11 (H11):** Individuals who have experienced a case of COVID-19 in their household are more likely to receive help from people outside their family.
**Hypothesis 12 (H12):** Individuals who have experienced a case of COVID-19 in their household are more likely to help others outside their family.
**Hypothesis 13 (H13):** Individuals who have experienced financial hardship due to COVID-19 are more likely to receive help from people outside their family.
**Hypothesis 14 (H14):** Individuals who have experienced financial hardship due to COVID-19 are more likely to help others outside their family.

## Data and Measures

Our data come from an Internet survey of 4,234 Canadians and Americans, fielded between August 25 and August 31, 2020. The survey, designed by a consortium of researchers at Western University, asked respondents a broad range of questions relating to the COVID-19 pandemic. The survey was available in English in the United States, and in English and French in Canada. The sample was provided by Leger Opinion and (for 18 percent of the American sample) DISQO. The sample was created using quotas for country (equal sample size for each), as well as census-based quotas to ensure representativeness by age, gender, and region (six regions in Canada, eight in the United States). Representativeness was further supported by weighting responses on all quota criteria. For each model, we rely on listwise deletion which deletes all observations (in this case, individuals) that have one or more missing values on the independent variables.^3^ In doing so, the original sample of 4,234 observations was reduced by about 18 percent: Model 1 has 3,510 observations, Model 2 has 3,511, and Models 3 and 4 have 3,471.

Our dependent variables are receiving and giving practical help outside the family. We operationalized these using a pair of survey questions: “Which of the following things have you done to help people who are not members of your own family during the COVID-19 pandemic?”; and “Which of the following things have people (other than members of your own family) done to help you and your household during the COVID-19 pandemic?” For both questions, we further noted, “Do not include paid services, or activities in your own household.” Respondents chose from the same list of options for both questions, with instructions to check all response categories that applied. The response categories were “cooked and/or delivered meals”; “run errands (such as grocery shopping or a trip to the pharmacy)”; “lent or given household supplies or tools”; “lent or given money”; “helped with household maintenance (such as garden work or chores)”; “provided childcare”; “other (please specify)”; and “none”; and “don’t know/prefer not to answer.” For our analysis, we constructed binary measures for both variables by assigning a “1” for respondents who reported giving (or receiving) at least one kind of help, and “0” for respondents who reported not giving (or receiving) any help.

Research that asks individuals to report on socially desirable activities frequently suffers from measurement bias, since respondents are motivated to over-report such activities (Brenner and DeLamater 2016). Our survey design attempted to limit the scope for measurement bias in two ways. First, we asked respondents to select from a list of concrete helping acts, rather than asking a broader question about helpfulness. Second, we asked about what respondents had done to help others prior to asking about receiving help, since asking first about received help might have accentuated respondents’ desire to report having helped others.

To assess community ties, we asked respondents about their connections with neighbors, community organizations, and friends. Our “neighbors” variable is based on the survey question, “How many of your neighbors do you know personally?” Respondents could choose from four categories (none, a few, most, or all). We re-coded this into three categories, combining “most” and “all” to ensure a more equitable balance of responses across categories without any substantial loss of information. Our measure of connectedness to community organizations was built from this question: “In the past 12 months, were you a member or participant in any of the following types of groups, organizations or associations? (check all that apply)” and provided a list of 13 response categories. From this list, we identified nine categories that represent locally based organizations.^4^ We then created a binary variable by coding all respondents who checked one or more of these nine categories as members of community organizations, as all others as non-members. Finally, our “friends” measure is also a binary (yes/no) variable, based on responses to the question, “Do you have any close friends who live in the same city or local community as you?”

To ensure that our measures of community ties are empirically distinct from each other, we conducted a diagnostic correlation test of responses in our survey data. All three variables are weakly and positively correlated. Community organization membership and having a close friend in the community have a correlation score of 0.14; community organization membership and knowing one’s neighbors have a correlation score of 0.12; and knowing one’s neighbors and having a friend in the community have a correlation score of 0.18.^5^ The positive, weak correlations support our theoretical contention that these forms of community ties are related but distinct.

Our measures of personal crisis are based on the questions “Since March, have you or someone in your household had COVID-19?,” and “Are you feeling financial hardship due to COVID-19?” The former is a simple binary measure. The latter has five response categories, ranging from “no hardship” to “serious hardship.” We re-coded these responses to create a binary (yes/no) variable, distinguishing those who had experienced at least some COVID-related financial hardship from those who had experienced none.

## Models and Controls

To empirically test our hypothesized relationships, we use survey-weighted generalized linear models. Given that our observations are nested within countries, the assumption of independence is violated. Since our analysis focuses on aggregate patterns, we want to control for country-level context. To do this, we use country-level fixed effects to capture unobserved heterogeneity in the data. Since we have two dependent variables (giving help and receiving help), we test our hypotheses using paired models. We use Models 1 and 2 to test hypotheses H1 to H6 (concerning community ties) and hypotheses H11 to H14 (concerning need and help). In Model 1, the dependent variable is receiving help; in Model 2 it is giving help. Both models include all five independent variables, such that they serve as controls for each other in the analysis. Models 3 and 4 are the same as 1 and 2, except that one of our two dependent variables is added to each model and used to predict the other. We use Models 3 and 4 to test hypotheses H7 to H10 concerning social exchange and to perform a robustness test on H11 to H14.

In addition to the five independent variables, our models also include a range of controls. Among them are three standard demographic variables (*household income, gender*, and *level of education*), as well as some others that we expect may influence helping behaviors. Since literature on disaster resilience suggests that levels of social trust may have independent effects on social behavior in crisis situations (Kyne and Aldrich 2020), we include a measure of *generalized trust*, which we assess through a question about whether most people can be trusted; and a measure of *trust in neighbors*, which we assess through a question about whether respondents trust most, many, a few, or none of the people in their neighborhood.

Second, we control for *age*. There are some reasons to expect that age might have an independent impact on patterns of helping even when controlling for community ties. Prior to the introduction of vaccines, adults aged 65 or older were much more vulnerable to serious illness if they contracted the virus. This vulnerability might lead older individuals to reach out more for help; it might also lead others in the community to be altruistically helpful to older adults. However, there is also evidence that older adults have faced more challenges in asking for help during the COVID-19 pandemic due to factors such as limited competence with computer technologies (Gauthier et al. 2021) or a fear of physical contact with others (Tyrrell and Williams 2020).

Third, we control for *self-rated health status* since we might expect overall health (not necessarily related to the pandemic) to influence need, and therefore patterns of helping. Finally, there is a substantial literature on the relationship between built form and social ties, with some studies suggesting that high-density living arrangements are marked by weaker and more segmented social networks among neighbors (Freeman 2001; Knies 2013; McCabe 2013). We therefore control for *built environment* by re-coding responses to a question about the type of dwelling in which respondents live into a binary (high/low density) variable. Summary statistics for all our variables are included in online Appendix B.

## Descriptive Statistics: Prevalence, Types and Sources Of Practical Help

**Figure 2.**
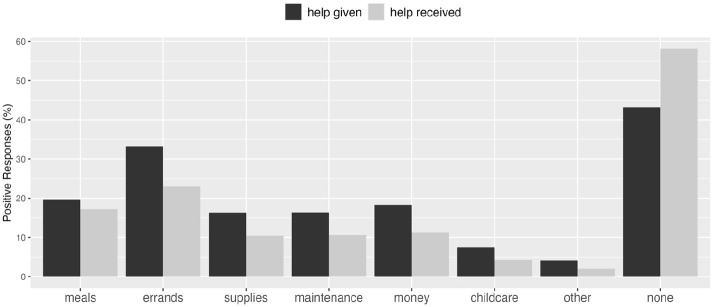
Unpaid help outside the family during the COVID-19 pandemic. *Note:* Survey N = 4,234 ‘Don’t know/refused to answer’ recoded as missing. Non missing N for help given = 4,077. Non-missing N for help received = 4,085.

Before we turn to our model results, let us briefly look at the descriptive data. Figure 2 shows percentage frequencies for giving and receiving help. Helping behaviors outside the family during the pandemic were widespread among our respondents. Twenty percent of respondents cooked or delivered meals to others, and 17 percent had meals cooked or delivered to them or their household; nearly one-third of respondents (33 percent) ran errands for others, while 23 percent reported that someone ran errands for them. Three other categories had positive response rates of 10 percent or more for both giving and receiving.

Overall, only 43 percent of respondents said they had not helped others outside their family, whereas 58 percent of respondents said they had not received any help from others outside their family. There are at least two possible reasons for the discrepancy between reported levels of giving and receiving help, which holds across every response category. One is that help-giving is targeted toward a smaller number of people and households in particular need. The other is that respondents over-reported giving help, despite our survey design safeguards. Since we did not ask respondents how many people they had helped (or been helped by), we cannot tell which of these explanations, if either, is valid.

**Figure 3.**
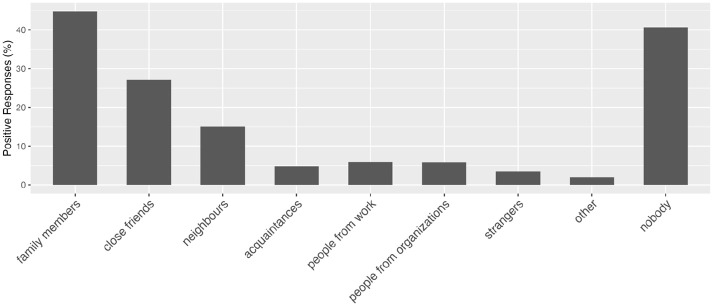
Sources of practical help during the COVID-19 pandemic. *Note:* Survey N = 4,234 ‘Don’t know/refused to answer’ recoded as missing. Non missing N = 4,120.

We also asked survey respondents, “Who has given practical help to you and your household during the COVID-19 pandemic?” Figure 3 shows the responses. Since the data include help from family members, the percentage of respondents who reported not getting help from anyone (41 percent) is lower than in Figure 2. Figure 3 thus confirms the importance of family members as sources of help in crisis conditions. However, it also makes clear that relationships with close friends and neighbors are common sources of help as well. The prevalence of different sources of help, however, does not necessarily determine their *importance* to individuals. Some kinds of relationships may be uncommon, but highly valuable to those who are in them. We now turn to our analysis of the significance of different kinds of community ties for helping behaviors.

## Results of Hypothesis Tests

### Community Ties and Helping Behaviors

We start by examining the association between different kinds of community ties and helping behaviors using Model 1 (where receiving help is the dependent variable) and Model 2 (where giving help is the dependent variable). We present the full results in online Appendix A (available at https://doi.org/10.5683/SP3/UB9P9P). However, since interpretation of the logistic regression coefficients presented in model outputs is not intuitive, we communicate model results by generating predicted probabilities using the average marginal effects approach.^6^

**Figure 4.**
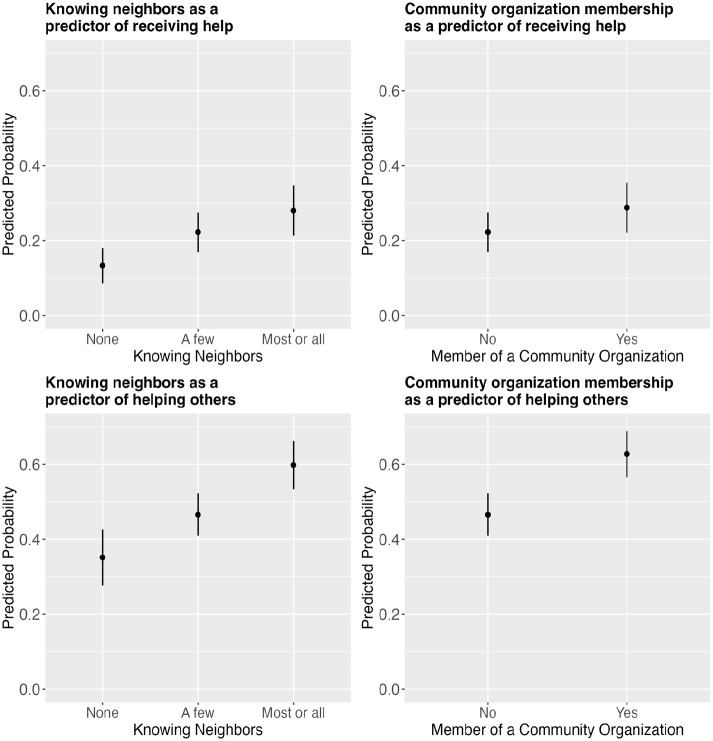
Knowing neighbors, organization membership, and helping behaviors.

Figure 4 presents predicted probability results of our tests for hypotheses H1 through H4, which concern “weak” community ties among neighbors and through community associations. When controlling for all the other variables discussed above, we find a statistically significant (*p* < .01) positive relationship in all four cases. Respondents who do not know their neighbors have a 13 percent predicted probability of receiving help during the COVID-19 pandemic, whereas this rises to 22 percent for those who know a few neighbors, and 28 percent for those who know most or all of their neighbors. The predicted probability of receiving help from non-family members is 22 percent for those who are not members of community organizations, but it rises to 29 percent for those who are members. Our results thus support hypotheses H1 and H3, suggesting that during the early months of the COVID-19 pandemic, community associations and neighbors both provided significant access to help.

Our results likewise support hypotheses H2 and H4, which test the significance of weak ties for help-giving. Among those who know none of their neighbors, the predicted probability of helping others during the pandemic is 35 percent, whereas when a person knows most or all of their neighbors, the predicted probability of helping others is 60 percent. Meanwhile, the predicted probability of helping others in the community is 47 percent for those who are not members of community organizations, but 63 percent for those who are members.

**Figure 5.**
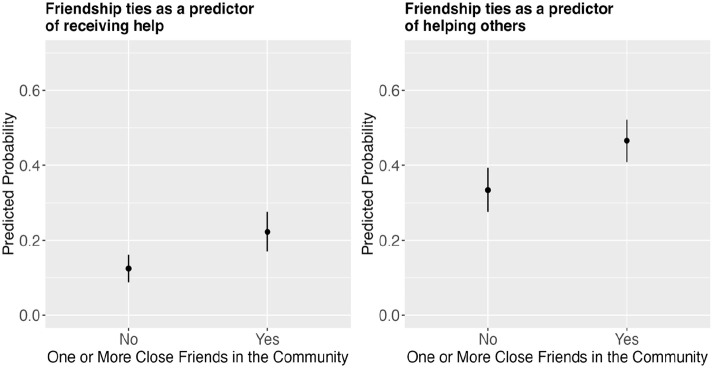
Friendship ties and helping behaviors.

Turning now to the “strong ties” of friendship, Figure 5 presents predicted probability results for hypotheses H5 and H6. Once again, both hypotheses are supported with statistically significant (*p* < .01) positive associations. For individuals who do not have close friends in the community, the predicted probability of receiving help is 12 percent; this rises to 22 percent for those who do have one or more close friends living in the community. The predicted probability of giving help is 33 percent for those who do not have close friends in the community, and 47 percent for those who do.

Our findings thus support the proposition that community ties are significant sources of access to practical help in crisis conditions; the corollary of this proposition, that community ties are conduits for help-giving, is also supported. Our results also support the idea that different kinds of community ties are complementary, since each kind of ties individually affects the likelihood of helping behaviors when controlling for the others. Finally, they suggest that both weak ties (neighbors and community associations) and strong ties (close friends) are both important conduits of access to practical help.

To further investigate the relative importance of our three community ties variables as predictors of giving and receiving help, we use a simulation approach. Using 2,500 simulations, we generate an average marginal effect (AME) distribution for each variable while holding all other variables at their observed values. We then compare the distributions of the simulated AMEs for each variable.^7^ We find that knowing one’s neighbors is the strongest predictor of both giving and receiving help. Having a close friend in the community is a stronger predictor of receiving help than community organization membership; conversely, community organization membership is a stronger predictor of giving help than having a close friend.

### Social Exchange and Helping Behaviors

**Figure 6.**
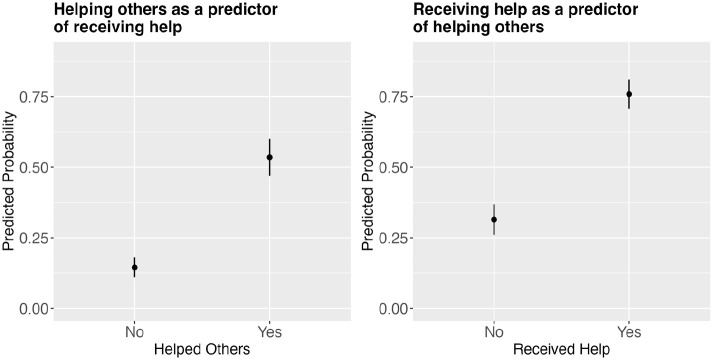
Social exchange and helping behaviors.

We now turn to our proposition that practical help is structured by social exchange. To test hypotheses H7 and H8 we use Models 3 and 4, which introduce giving help as a predictor of receiving help and vice versa, while retaining all variables from Models 1 and 2. Full results are again presented in online Appendix A. We find remarkably strong, statistically significant (*p* < .01) support for both hypotheses (Figure 6). In the left plot, we see that individuals who did not provide help to non-family during the pandemic had only a 15 percent predicted probability of receiving help from non-family, whereas those who reported helping others had a 54 percent probability of receiving help. The right plot shows that those who reported they did not receive help had only a 31 percent predicted probability of helping others, whereas this rose to 76 percent for those who had received help.

There thus is a very strong, two-way positive association between giving and receiving help in our data, which suggests that social exchange is an important structuring element of helping behaviors outside of the family in the COVID-19 context. We cannot comment on the relative importance of reciprocal vs. generalized exchange, although—as we noted earlier—we fully expect that both are at work in structuring helping behaviors. We should also note that since our empirical measures of helping are restricted to the COVID-19 pandemic period, our results do not capture social exchange processes that occurred before the onset of the pandemic.

We also use Models 3 and 4 to test hypotheses H9 and H10, regarding social exchange as a mediator between community ties and helping behaviors. If community ties provide access to help because they are venues in which individuals practice social exchange, then introducing social exchange into our models should eliminate the effects of community ties on helping behaviors. The full model results in online Appendix A show that the strength of the association for all six relationships outlined in hypotheses H1 through H6 decreases once giving help is introduced as a predictor of receiving help and vice versa. However, in five out of six cases statistically significant relationships remain, which suggests partial, rather than full mediation.

To further investigate this, we perform causal mediation analysis on Models 3 and 4 using the approach outlined by Imai, Keele, and Tingley (2010). The results are presented in Table 1. They confirm that the association between all three community ties variables and the likelihood of giving and receiving help is *partially mediated* by social exchange. The strength of partial mediation varies, with the average proportion mediated ranging from 0.173 for the relationship between community organization membership and giving help, to 0.72556 for the relationship between community organization membership and receiving help. The mediating role of social exchange also differs depending on the kind of community ties. For members of community organizations, the increased likelihood of receiving help is largely contingent on help-giving, whereas those who know their neighbors and/or have close friends in the community are significantly more likely to get help even if they do not give it. Conversely, members of community organizations are more likely to give help even if they have not received it, compared with those who know their neighbors and/or have close friends in the community.

**Table 1.** Mediation Analysis: Social Exchange, Community Ties, and Helping Behaviors.

Model	Mediator	Community ties variable being tested	Average mediated effect	Average direct effect	Total effect	Average proportion mediated
Model 3 (DV: received help)	Gave help	Community organization membership	0.05301	0.02005	0.07306	0.72556
		Neighbors	0.0308	0.0914	0.1222	0.2519
		Friends	0.0551	0.1152	0.1702	0.3235
Model 4 (DV: gave help)	Received help	Community organization membership	0.0245	0.1172	0.1417	0.173
		Neighbors	0.0505	0.0324	0.0829	0.6091
		Friends	0.0700	0.0674	0.1373	0.5095

*Note.* All results are statistically significant *p* < .001. DV = dependent variable.

Our results, then, provide partial support for our mediation hypotheses, H9 and H10. We see two plausible (and not mutually exclusive) explanations for partial mediation. First, since our survey questions asked respondents about helping *during* the COVID-19 pandemic, our data do not capture how pre-pandemic social exchange processes shape pandemic-era helping behaviors. For instance, an individual who received help from neighbors or close friends during the pandemic but did not give it may have done so before the pandemic. Second, it is likely that other social mechanisms, such as solidarity-based altruistic helping, also motivate help-giving in a community crisis. Specifically, our mediation results suggest that altruistic help-giving may be particularly prevalent among those who are active in community associations, a result that aligns with established findings in the literature on volunteerism and helping (Jackson et al. 1995).

### Need and Helping Behaviors

**Figure 7.**
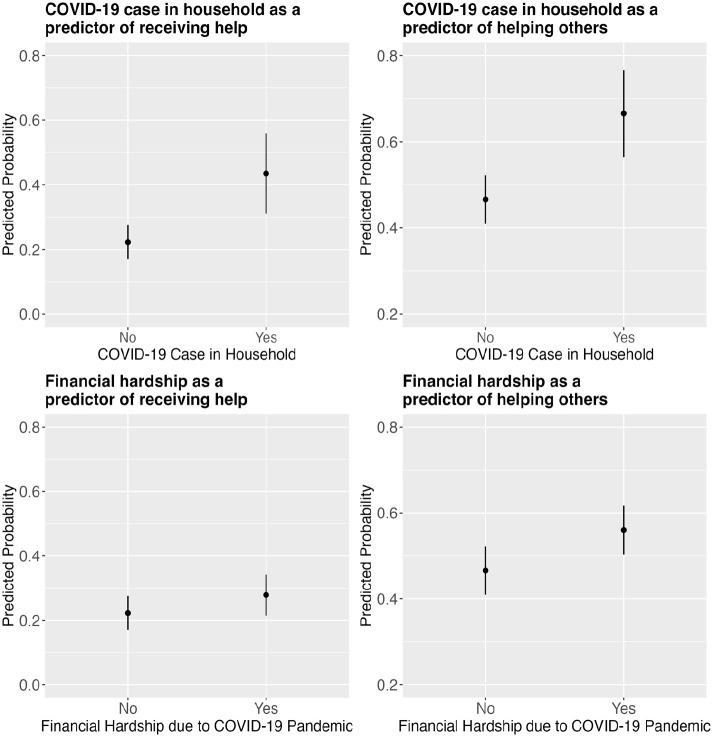
Need and helping behaviors.

To test hypotheses H11 to H14, concerning the relationship between need and helping behaviors, we first return to Models 1 and 2. Recall that we theorize that those in extraordinary need are more likely to receive help—whether as a result of asking, or as a result of being seen to be in need by others—but that insofar as helping is structured by social exchange, these individuals will also be more likely to give help to others. As seen in Figure 7, our results are consistent with this account, with statistically significant (*p* < .01) positive relationships in all four cases. Those who had experienced a COVID-19 case in their household were much more likely than others to receive help (H11), with the predicted probability rising from 22 to 44 percent. Those who experienced financial hardship due to the pandemic were also more likely to receive help (H13), with the predicted probability rising from 22 to 28 percent.^8^ Notably, those who had experienced a case of COVID-19 in the household were more likely than others to *give* help (H12), with the predicted probability rising from 47 to 67 percent, as were those who had experienced financial hardship (H14—predicted probability rises from 47 to 56 percent).

Our results for Models 1 and 2 are thus consistent with a social exchange account of helping behaviors in crisis. Turning now to Models 3 and 4—which introduce the helping variables as predictors and controls—we see that in most cases the association between acute need and helping behaviors remains but is reduced (see online Appendix A). The exception here is the relationship between financial hardship and receiving help, which is no longer statistically significant when controlling for giving help. This suggests that receiving help when in financial crisis (H13) may be fully contingent on the ability to provide help to others during the pandemic. In addition, while Models 2 and 4 both show a significant relationship between experiencing COVID-19 and giving help to others, further investigation reveals that this relationship is moderated by community organization membership, such that having had COVID-19 in the household is only a significant predictor of providing help for individuals who are also members of a community organization.^9^

### Additional Results

Our models also produced several results beyond our hypothesis tests that are worth commenting on (see online Appendix A for details). First, counter to the expectations of literature on social capital and social resilience (Kyne and Aldrich 2020), we found that neighborhood trust and generalized trust are, for the most part, *not* significant predictors of helping behaviors. Second, Models 1 and 3 both identified a statistically significant (*p* < .01) negative relationship between age and help received. This is consistent with findings from prior qualitative research (Gauthier et al. 2021; Tyrrell and Williams 2020) that older adults had trouble accessing interpersonal help during the early months of the COVID-19 pandemic due to limited computer skills and/or a fear of physical contact. However, results from Model 1 suggest that people are significantly less likely to give and receive help in the community after age 45, not after age 65, which is not easily explained using the findings from prior research. An alternative explanation might be that older adults rely more on family (and thus less on others in the community) for support than younger adults.

Finally, Model 3 identified a very weak but statistically significant (*p* < .01) positive relationship between household income and giving help, and Model 4 shows a weak but statistically significant negative relationship between household income and receiving help. This suggests that there was a limited socially redistributive element to helping behaviors in the early months of the COVID-19 pandemic. Intriguing as this finding is, given that our composite measure of practical help includes various activities (ranging from running errands to lending money) that might be expected to vary in relative importance by household income, only a more disaggregated analysis of help would give us insight into the substantive significance of this redistributive tendency.

## Discussion and Conclusions

Overall, our results provide robust empirical support for our model of factors that shape access to practical help outside the family in the context of a community-wide crisis. Our work extends existing understanding in several ways. First, it allows us to move beyond general assertions in the disaster resilience literature that community ties matter for individual well-being. Focusing on three specific kinds of community ties—neighborhood, associational, and friendship ties—we find that each of them was a significant avenue of access to practical help in the COVID-19 context when controlling for the others. Our findings thus provide broad-based evidence for Wellman and Wortley’s (1990) insight, that different kinds of community ties are complementary elements of an ensemble of social connections that individuals can use to access help when in need.

Second, we articulate and empirically substantiate an exchange-based view of help in crisis conditions. From this perspective, giving and receiving are two aspects of the broader phenomenon of helping behaviors—the reciprocal and generalized exchange of help through social relationships. The results of our empirical tests support this perspective, in that we find a strong direct association between giving and receiving help when controlling for a wide range of other factors, and we find that social exchange significantly mediates all relationships between community ties and helping.

Finally, our results corroborate earlier findings in the social support and disaster resilience literatures that those in extraordinary need receive more help than others; but they also suggest that in certain cases, those in extraordinary need *give* more help than others, which is consistent with an exchange-based perspective on helping. This does not necessarily mean, of course, that those in crisis help others more *when* they are facing extraordinary need. We also do not know who individuals in need gave help to, or what specific kind of help they gave. What we can say is that it appears that across North America, practical helping beyond the family during the early months of the pandemic was significantly grounded in social exchange processes.

Our results raise numerous questions for future research, some but not all of which could be addressed through further analysis of our data. We will highlight five such questions by way of closing. First, do the social dynamics of practical help differ across different forms of help? The measure of practical help used in this article is a composite one that includes a variety of activities, ranging from cooking meals and running errands to lending tools and money. It is likely that different kinds of practical help draw on different community ties, and some kinds of helping may be more strongly anchored in social exchange than others. The present analysis may thus elide significant differences in the dynamics of different kinds of practical help, something that could be investigated by disaggregating our dependent variable into its component parts.

Second, do individuals who have multiple kinds of community ties give and/or receive more help than those who have only one kind of tie? Our theoretical model suggests that this should be the case, but we have not yet tested this relationship empirically. Third, how well do our findings apply beyond the COVID-19 context? A pandemic obviously differs from a natural disaster in terms of the kinds of disruptions faced by individuals and households, and therefore the kind of practical help that is likely to be needed. However, we see no reason a priori to expect that our findings regarding community ties and the exchange basis of help outside the family would not hold in disaster or other community-wide crisis settings. Furthermore, while we have focused on crisis situations, community ties and social exchange may be equally important for access to help outside the family in non-crisis times. Large-N empirical work in a variety of other crisis and non-crisis settings would be needed to explore the empirical reach of our findings.

Fourth, how do helping behaviors outside the family interact with helping behaviors within the family? While we collected detailed data only on non-family patterns of helping, we know that 42 percent of our respondents received help both from family members and non-family members. Our data do not capture any social exchange that may take place across the family/non-family boundary, so our analysis excludes a potentially significant dimension of overall social exchange. Including within-family help in future studies would address this lacuna and would also enable comparison of the prevalence of exchange dynamics in family and non-family helping behaviors.

Finally, how is practical help in crises influenced by helping behaviors in non-crisis times? Our model assumes that the community ties individuals use to access help in a crisis, as well as the social exchange processes that structure helping behaviors, are largely in place before the crisis occurs. This “stock” of previously developed social resources is what individuals can then draw on in times of extraordinary need. However, our survey questions did not ask about helping behaviors prior to the pandemic, so we cannot confirm this. To advance our understanding would involve diachronic survey research that spans non-crisis and crisis times, which might shed light on how community ties developed in a non-crisis context influence patterns of helping in times of crisis, when practical help in the community can be a lifeline for the many individuals and households who face extraordinary challenges and needs.

## Supplemental Material

sj-docx-1-cty-10.1177_15356841231159370 – Supplemental material for Somebody to Lean On: Community Ties, Social Exchange, and Practical Help during the COVID-19 Pandemic Click here for additional data file. Supplemental material, sj-docx-1-cty-10.1177_15356841231159370 for Somebody to Lean On: Community Ties, Social Exchange, and Practical Help during the COVID-19 Pandemic by Martin Horak and Shanaya Vanhooren in City & Community

## Notes

1. Stephenson et al. (2020), Scholars Portal Dataverse, V1.

2. Calculated from responses to the question: “Who has given practical help to you and your household during the COVID-19 pandemic?”

3. While listwise deletion can cause bias if missingness is systematic, having compared demographics of the sample before and after listwise deletion, we have no reason to believe that systematic bias was introduced. We also have no reason to believe that listwise deletion is causing inefficiency, given the healthy-sized N (minimum of 3,471 observations in all models) that remains after listwise deletion.

4. The nine categories are sports or recreational organization (such as a soccer league, health club or golf club); cultural, educational, or hobby organization (such as a theater group, book club or games club); a religious-affiliated group (such as a choir or youth group); school group, neighborhood, civic, or community association (such as a parent-teacher association, block parent group, or neighborhood watch); a service club (such as Kiwanis, Optimist Club or Legion); a senior’s group; a youth organization (such as Scouts, Guides, or YMCA/YWCA); ethnic or immigration association or club; and a co-operative organization (such as a housing co-op or community garden).

5. For the purposes of the correlation test, we recoded the neighbors variable as binary (where 1 = know none of neighbors and 2 = know some, most, or all of neighbors).

6. When generating predicted probabilities for non-linear limited dependent variable models, the values at which the other variables are set will influence the size of the effect. The average marginal effects (AME) approach is preferable because it uses the actual values taken on by each observation (Hanmer and Kalkan 2013). We also used expected proportional reduction in error (ePRE) measures to understand how much better we did at predicting the values of the dependent variable compared with using a null model. The ePRE measures for our models are as follows: Model 1—0.12 (12 percent); Model 2—0.11 (11 percent); Models 3 and 4—0.25 (25 percent).

7. Due to space constraints, we do not discuss the full simulation results here. They are available on request from the authors.

8. Since our help variables are composed of multiple specific items, we also correlated the COVID-19 and financial need variables with specific help received items to see which items drive the overall correlations. We find that having had COVID-19 in the household is associated with a strong increase in help received with meals, supplies, and errands; having experienced financial distress is associated with a strong increase in financial help and help with meals. These intuitive results support the face validity of our item measures.

9. An anonymous reviewer pointed out that the relationship between the community ties variables and receiving help might be moderated by need, since having an acute need might make community ties more valuable. We explored this possibility using interaction terms. We did not find any statistically significant interactions between our acute need variables (COVID-19 in the household and financial hardship) and community ties variables in any of the receiving help models. For the giving help models, and we did find a statistically significant interaction between having a COVID-19 case in the household and community organization membership.We cannot definitively explain this result, but it may be that the relationship posited in H13 is contingent on social exchange practices specific to community organizations; or that certain individuals are more likely to be community organization members, help others, *and* have experienced COVID-19 due to the influence of some other variable not captured in our models.
