# Supplementary material for: Somebody to Lean On: Community Ties, Social Exchange, and Practical Help during the COVID-19 Pandemic
Source: City Community. 2023 Mar 22:15356841231159370. doi: 10.1177/15356841231159370 (PMC10034562; doi:10.1177/15356841231159370)
Supplement: sj-docx-1-cty-10.1177_15356841231159370 – Supplemental material for Somebody to Lean On: Community Ties, Social Exchange, and Practical Help during the COVID-19 Pandemic [file sj-docx-1-cty-10.1177_15356841231159370.docx]

***Abstract***

During a community-wide crisis, practical help from others in the community can allow individuals to manage a variety of extraordinary household needs. In this paper, we synthesize insights from research on disaster resilience, social support, social networks and social exchange into a theoretical model of factors that shape individual access to help beyond the family. We suggest that community ties – local neighbourhood, associational, and friend relationships – are significant avenues for accessing help, and that helping behaviours in the community are structured by social exchange. We test this model in the early months of the COVID-19 pandemic, drawing on a survey of 4,234 Canadians and Americans. We find that all three kinds of community ties significantly increase the likelihood of receiving and giving help; that there is a strong, positive two-way correlation between giving help and receiving help; that relationships between community ties and helping behaviors are mediated by social exchange; and that individuals in extraordinary need tend to both receive and give more help than others. Our findings provide broad-based evidence for the importance of local social ties and social exchange processes in structuring access to practical help in times of extraordinary need.
